# Longitudinal Relations Between Internet Gaming Disorder Symptoms, Depressive Symptoms, and Hikikomori Symptoms Among Young Gamers – Random Intercept Cross-Lagged Panel Model With Contextual Factors

**DOI:** 10.1007/s10964-026-02352-7

**Published:** 2026-04-15

**Authors:** Xingcan Ye, Ted C. T. Fong, Paul S. F. Yip

**Affiliations:** 1 Department of Social Work and Social Administration, The University of Hong Kong, Hong Kong, Hong Kong; 2 Research Hub of Population Studies, The University of Hong Kong, Hong Kong, Hong Kong; 3 Social Science Research Centre, The University of Hong Kong, Hong Kong Hong Kong, Hong Kong; 4 The HKJC Centre for Suicide Research and Prevention, The University of Hong Kong, Hong Kong, Hong Kong

**Keywords:** Random intercept cross-lagged panel model, Comorbidity, Depressive symptoms, Developmental difference, Internet gaming disorder symptoms, Hikikomori

## Abstract

Despite the high comorbidity of internet gaming disorder symptoms, depressive symptoms, and hikikomori symptoms (severe social withdrawal), their longitudinal relations remain underexplored, especially whether they directly exacerbate one another over time or stem from shared underlying vulnerabilities. The present study used random intercept cross-lagged panel models to differentiate longitudinal within-person effects from stable between-person associations between internet gaming disorder, depressive, and hikikomori symptoms. The sample comprised 1560 gamers (45.3% female, M_age_ = 23.38, SD = 3.81 years) in Hong Kong who played internet games for at least three hours per week. Participants were divided into two age groups: 875 youth (43.20% female, M_age_ = 20.52, SD = 2.38 years) and 685 adults (47.88% female, M_age_ = 27.03, SD = 1.42 years). Including family and in-game contextual factors at the between-person level, two random intercept cross-lagged panel models across age groups were constructed and revealed developmental differences. Among youth gamers, hikikomori symptoms positively predicted depressive symptoms, and internet gaming disorder symptoms marginally predicted higher hikikomori symptoms. No significant within-person effects were found in adult gamers. Internet gaming disorder, depressive, and hikikomori symptoms showed significant positive between-person associations, and the escapism motive was a common risk factor in both youth and adult gamers. Playing specific game genres was associated with comorbidity among youth gamers, whereas the disruptor trait was associated with comorbidity among adult gamers. Lower family satisfaction contributed to depressive and hikikomori symptoms. These results highlight that longitudinal within-person relations might explain the comorbidity among internet gaming disorder, depressive, and hikikomori symptoms in youth gamers, whereas stable between-person associations may account for their co-occurrence in adult gamers. Clinical interventions should be developed for the sequential symptom cascade in youth and target shared underlying vulnerabilities to reduce comorbidity in adult gamers.

**Supplementary Information:**

The online version contains supplementary material available at 10.1007/s10964-026-02352-7.

## Introduction

Internet gaming disorder denotes a condition in which excessive engagement in internet games causes significant impairment or distress in a person’s life (American Psychiatric Association, 2013). Adolescents and young adults report a higher prevalence of internet gaming disorder at 9.9% compared to other age groups (Gao et al., 2022). Additionally, internet gaming disorder has been found to co-occur with other psychosocial problems such as depressive symptoms and hikikomori, with comorbid prevalence rates of 32%-62.3% and 28.5%-48.4%, respectively (Fong et al., 2023; Ostinelli et al., 2021). The high comorbidity of internet gaming disorder, depressive, and hikikomori symptoms can affect the effectiveness and long-term outcomes of mental health treatments. To identify which issue may be the primary problem, longitudinal research on internet gaming disorder, depressive, and hikikomori symptoms integrally is needed. Current research on three constructs is limited and cross-sectional (Fong et al. 2024a, b), so the underlying directionality remains unclear. Furthermore, few studies have yielded mixed results regarding the longitudinal within-person relations between internet gaming disorder and depressive symptoms without considering factors that could explain stable between-person associations (Hygen et al., 2020; Wei et al., 2025; Zhang et al., 2025). There is a lack of research on the longitudinal relations between hikikomori and others. To fill these gaps, this study used the random intercept cross-lagged panel model (RI-CLPM) to analyze within-person longitudinal relations among internet gaming disorder, depressive, and hikikomori symptoms, considering family and in-game contextual factors that might influence between-person associations.

### Theoretical Basis

Several theories explain the bidirectional relations between internet gaming disorder symptoms and psychosocial problems. The model of compensatory internet use posits a directional link from psychosocial problems to internet gaming disorder symptoms; that is, over-dependence on internet games due to psychosocial problems may increase the risk of developing internet gaming disorder symptoms (Kardefelt-Winther, 2014). Specifically, young people with depressive symptoms may cope with negative emotions through online games, while individuals with hikikomori symptoms compensate for social needs that cannot be fulfilled by their limited social activities and dysfunctional interpersonal skills in real life. Conversely, social displacement theory suggests that internet gaming disorder symptoms can lead to increased depressive and hikikomori symptoms because excessive gaming may occupy individuals’ real-world lives and impair their functioning, thereby inducing heightened negative emotions and reduced social interactions (Kowert et al., 2014). Beyond the longitudinal relations, the common cause hypothesis argues that internet gaming disorder symptoms share risk factors with depressive and hikikomori symptoms, rather than being a cause or consequence (Hygen et al., 2020). Regarding the longitudinal relations between depressive and hikikomori symptoms, the interpersonal theory of depression supports a bidirectional process in which depressed individuals may express maladaptive social behaviors, such as withdrawal from social situations (Hames et al., 2013). In turn, impaired social functioning, feelings of social isolation, and unfulfilled social needs increase the risk of future depression.

### Internet Gaming Disorder and Depressive Symptoms

Previous longitudinal studies have shown mixed findings regarding the directional relations between internet gaming disorder and depressive symptoms. Some studies using cross-lagged panel models (CLPM) found that depressive symptoms predicted later risk of internet gaming disorder (Dang et al., 2024; Teng et al., 2021), whereas others suggested that internet gaming disorder symptoms worsen depressive symptoms (Wang et al., 2022). Still, other research revealed bidirectional relations at both the overall and symptom levels (Jeong et al., 2019; Ye et al., 2025). These inconsistent results may arise from limitations of cross-lagged models, which cannot differentiate within-person effects from stable between-person differences. By adding random intercepts to account for trait-like between-person differences, the RI-CLPM more accurately captures within-person effects over time. Although a few studies have used RI-CLPM, they have produced mixed results regarding within-person effects. For example, two RI-CLPM studies found that the longitudinal relation from depressive to internet gaming disorder symptoms was present only in children, not in early adolescents (Hygen et al., 2020; Zhang et al., 2025). The other study found opposite directional relations; that is, the within-person effect of internet gaming disorder symptoms on future anhedonia, and depressed mood on internet gaming disorder symptoms (Wei et al., 2025). The inconsistency may result from differences across age groups and from time-invariant confounding factors that influence individuals’ propensities for internet gaming disorder and depressive symptoms. Such cofactors may be related to trait-like between-person differences and may indirectly affect longitudinal relations.

### Internet Gaming Disorder and Hikikomori Symptoms

Hikikomori, characterized by prolonged social withdrawal, is a condition in which an individual withdraws from social participation across various life domains, such as school, work, leisure, and interpersonal relations (Kato et al., 2019). Previous cross-sectional research has shown that young gamers at moderate and high risk of internet gaming disorder report more severe hikikomori symptoms (Fong et al., 2023). Nonetheless, longitudinal research on their relations is scarce. One CLPM study found a cross-lagged path from hikikomori symptoms to internet gaming disorder symptoms, but no reverse relation (Calaresi et al., 2025). Since hikikomori may partly reflect individuals’ interpersonal problems and social vulnerabilities, existing studies on the longitudinal relations between internet gaming disorder and interpersonal problems can serve as an empirical foundation for the potential bidirectional relations. For instance, some studies found that social vulnerability increased the risk of internet gaming disorder one year later (Peeters et al., 2018; Zhuang et al., 2023), while others showed that internet gaming disorder symptoms were associated with more interpersonal problems later (Benjet et al., 2023; Teng et al., 2020). More longitudinal evidence is needed to examine the within-person effects over time between internet gaming disorder and hikikomori symptoms, thereby clarifying the antecedents and consequences.

### Depressive and Hikikomori Symptoms 

A recent scoping review found the co-occurrence of depressive and hikikomori symptoms in young people but highlighted that the directionality of their relationship is still unestablished (Pupi et al., 2025). Limited longitudinal evidence has shown contradictory findings on the directional mechanism between depressive and hikikomori symptoms. A two-wave CLPM study showed that hikikomori tendencies increased depressive tendencies among official workers (Kubo et al., 2024), while a three-wave RI-CLPM study identified the longitudinal within-person effect of depressive symptoms on hikikomori behaviors (Nonaka, Takeda, & Sakai, 2026). These two studies focused on relatively older samples with a wide age range (30 to 59 years; 20 to 64 years), whereas hikikomori mainly occurs and develops in adolescents and young adults during transitional stages (Teo & Fetters et al., 2015).

### Contextual Factors

Various contextual factors may influence the development of internet gaming disorder, depressive, and hikikomori symptoms among young people. Self-determination theory suggests that real-world (e.g., family) and in-game (e.g., game genres) contexts allow gamers to satisfy their psychological needs, which are essential for mental well-being (Ryan et al., 2006). A positive parent-child relationship and parental status (e.g., income) serve as protective factors against mental health problems (Liu et al., 2024; Nonaka & Sakai, 2021; Schneider et al., 2017). Besides, young gamers are affected by the environment and settings of online games. For example, playing different game genres has been linked to varying levels of internet gaming disorder and depressive symptoms (Na et al., 2017), and it has influenced the longitudinal relations between internet gaming disorder and depressive symptoms (Ye et al., 2025). Thus, considering both family and gaming contexts can help explain the co-occurrence of internet gaming disorder, depressive, and hikikomori symptoms.

### Developmental Differences

Youth under 25 years old are still pursuing academic achievements and going through biopsychosocial development, including hormonal and physical changes, difficulties with emotion regulation, high demands of peer relations, and altered self-concept (Rapee et al., 2019). The neurological plasticity unique to youth makes them more sensitive to change and more prone to psychopathology (Backes & Bonnie, 2019). In contrast, young adults aged 25–29 have completed their higher education, entered the workforce, and placed more emphasis on work in their daily lives (Hatano et al., 2024). They may encounter different life stressors and adult responsibilities. A meta-analysis concluded that the proportion of individuals with onset of any mental disorders before age 14 and 25 was 34.6% and 62.5%, respectively, indicating the instability of mental disorders in youth and the increased likelihood of interactions among mental disorders (Solmi et al., 2022). A prior study using RI-CLPM on internet gaming disorder and depressive symptoms has shown different relations between children and adolescents (Zhang et al., 2025). A recent scoping review claims that hikikomori is more likely to evolve in younger individuals (Pupi et al., 2025). These findings suggest that the longitudinal relations among internet gaming disorder, depressive, and hikikomori symptoms may differ across developmental stages.

## Current Study

Although previous studies have shown interrelations among internet gaming disorder, depressive, and hikikomori symptoms, no longitudinal research has examined these relations simultaneously. As two interdependent pathways to the development of internet gaming disorder in clinical settings, the comorbidity of internet gaming disorder symptoms with depressive and hikikomori symptoms should be studied together. Mixed findings regarding the directional relations between internet gaming disorder and depressive symptoms suggest potential age differences and roles of contextual factors. The lack of research on the longitudinal relations between hikikomori symptoms and both internet gaming disorder and depressive symptoms indicates a need for more longitudinal evidence. The present study aimed to use RI-CLPMs to explore within-person effects over time after separating stable between-person relations. Two RI-CLPMs, incorporating family and gaming characteristics as contextual factors, were developed to examine differences between youth and adult gamers. This study had six hypotheses. At the within-person level, there would be reciprocal relations between internet gaming disorder and depressive symptoms (Hypothesis 1), internet gaming disorder and hikikomori symptoms (Hypothesis 2), and depressive and hikikomori symptoms (Hypothesis 3). The longitudinal within-person effects were hypothesized to differ across age groups (Hypothesis 4). At the between-person level, it was expected that the random intercepts of internet gaming disorder, depressive, and hikikomori symptoms would be positively associated (Hypothesis 5). The stable between-person associations could be explained by family and gaming-related contextual factors (Hypothesis 6).

## Methods

### Participants and Procedures

The present study used a three-wave longitudinal design, with the sample drawn from an ongoing four-wave survey on mental health and internet gaming behaviors among young people in Hong Kong. Inclusion criteria of the participants were: (1) resided in Hong Kong in the past six months; (2) aged between 15 and 29 years ; (3) regular gamers who played online games for at least three hours per week in the past month. Recruitment was promoted through mass emails to undergraduate students at local universities and on Facebook, Instagram, and Twitter channels. Data collection began in May 2023 (*n* = 1560, T1), followed by a 6-month follow-up (*n* = 601, T2) and a one-year follow-up (*n* = 408, T3). The current sample included 875 youth gamers (56.09%) aged 15 to 24 years and 685 adult gamers (43.91%) aged 25 to 29 years.

Participants completed an online survey in Qualtrics and were informed of the study purpose, duration (15 min), voluntary participation, and confidentiality of their responses. They were assured that they could withdraw from the survey at any time. One out of five participants received a 100-HKD e-voucher at T1, one out of three received a 100-HKD e-voucher at T2, and every respondent received a 50-HKD e-voucher at T3.

### Measures

#### Internet Gaming Disorder Symptoms

The 9-item Internet Gaming Disorder Scale – Short Form (Lemmens et al., 2015) assessed the nine symptoms of internet gaming disorder based on the fifth edition of the Diagnostic and Statistical Manual of Mental Disorders (American Psychiatric Association, 2013). The nine symptoms include preoccupation, withdrawal, tolerance, loss of control, loss of interest, continuation despite harm, deception, escaping negative moods, and negative consequences due to gaming. Each symptom was rated on a scale from 1 (never) to 5 (very often) to indicate the severity of gaming behaviors over the past six months. An example item for continuation despite harm was “Do you continue playing online games even if it impairs your interpersonal relations?” The average score across the nine items was used in this study, with McDonald’s omega (ω) coefficients of 0.89 at T1, 0.90 at T2, and 0.91 at T3.

#### Depressive Symptoms

The 9-item Patient Health Questionnaire assessed participants’ depressive symptoms over the past two weeks (Martin et al., 2006). A sample item was “feeling emotional upset, depressed, and hopeless.” Each item was rated on a 4-point Likert scale (0 = “Not at all” to 3 = “Almost every day”), and these ratings were averaged to assess depressive symptoms (ω = 0.85 at T1, 0.89 at T2, 0.87 at T3).

#### Hikikomori Symptoms

The 5-item Hikikomori Questionnaire (Teo & Gaw, 2010) was used to assess hikikomori symptoms, which has demonstrated good validity among young gamers in Hong Kong (Fong et al., 2023). Participants were asked whether they endorsed the following symptoms: (1) staying at home most of the time; (2) avoiding social connections; (3) experiencing significant functional impairment or distress due to social isolation; (4) feeling anxiety and shame when staying at home; and (5) social isolation lasting over six months. The total number of symptoms was summed to measure hikikomori severity (ω = 0.71 at T1, 0.72 at T2, and 0.76 at T3).

#### Covariates

Regarding socio-demographic and family factors, participants reported their sex, age, and monthly family income (1: less than $10,000 HKD; 8: more than $70,000 HKD; with $10,000 HKD intervals), as well as their satisfaction with family life from 1 (extremely unhappy) to 5 (extremely happy) at T1.

Regarding gaming-contextual factors, participants reported whether they played each of the five game genres in the last month at T1: first-person shooter games, strategy games, multiplayer online battle arena games, massively multiplayer online role-playing games, and sports games. The response was coded as 1 if they played, and 0 otherwise.

Additionally, two gaming motives strongly associated with internet gaming disorder were measured. Participants responded to a single question, “playing online games to avoid real-world problems,” on a 5-point Likert scale to assess the escapism motive from the Motives for Online Gaming Questionnaire, which has demonstrated good validity (Wu et al., 2016). The Gamification User Types Hexad scale was used to assess disruptor traits, with good validity in Hong Kong (Fong et al. 2024a, b), and measures individuals’ tendency to break rules and disrupt systems in games (Tondello et al., 2019). A sample item was “I do not like following rules.” Four items rated on a 7-point Likert scale (1 = “disagree extremely” to 7 = “agree extremely”) were averaged, with a marginally acceptable ω at 0.64 for T1.

#### Data Analysis

Little’s Missing Completely at Random test and χ^2^/t-tests were used to examine the missing data in the present sample. Depressive symptoms followed missing completely at random assumption (*χ*^2^ = 7.12, *p* = 0.212), whereas internet gaming disorder (*χ*^2^ = 79.50, *p* < 0.001) and hikikomori symptoms (*χ*^2^ = 50.79, *p* < 0.001) did not. Missing data in the variables were handled using the full-information maximum likelihood (FIML) method under the missing-at-random assumption.

Configural, weak (constraining only factor loadings), and strong (constraining both factor loadings and item intercepts) measurement invariance models were analyzed to verify the stability of the latent structures of the main variables. A difference in the comparative fit index (ΔCFI) of less than 0.01 between two models indicates the longitudinal measurement invariance across time (Cheung & Rensvold, 2002). As shown in Table S2, longitudinal measurement invariance was maintained in this study (all ΔCFIs < 0.01).

The lavaan package in R was used to estimate the RI-CLPM on internet gaming disorder, depressive, and hikikomori symptoms (Rosseel, 2012). By introducing random intercepts, RI-CLPM separated stable and trait-like between-person effects from longitudinal within-person effects, allowing the capture of longitudinal relations between fluctuations in different variables over time at the within-person level (Hamaker et al., 2015).

The initial step was to calculate intraclass correlations (ICCs) for internet gaming disorder, depressive, and hikikomori symptoms. Larger ICCs indicate a higher likelihood of between-person differences and weaker interpretability of within-person variations. Generally, an ICC threshold of 0.5 suggests a need to differentiate between between-group and within-group effects (Bobak et al., 2018). The sample consisted of two age groups: youth (15–24 years old) and adult gamers (25–29 years old). To better examine the effect of developmental differences on longitudinal relations, RI-CLPMs were constructed across two age groups. Each age group began with a fully unconstrained model (M1) and sequentially added equality constraints on autoregressive effects (M2), cross-lagged effects (M3), and variances and covariances (M4) across time points. After selecting the most parsimonious model, socio-demographic, family- and gaming-contextual factors, and gaming motives were added as time-invariant predictors of the intercepts of internet gaming disorder, depressive, and hikikomori symptoms in the final model. A higher comparative fit index (CFI > 0.95) and Tucker-Lewis index (TLI > 0.95), a smaller Root Mean Square Error of Approximation (RMSEA < 0.08), a narrower confidence interval of RMSEA, and a decrease in AIC and BIC would suggest a better model fit (Hu & Bentler, 1999).

To verify the consistency and robustness of results with FIML imputation, the RI-CLPM on the sample without partial respondents (*n* = 408) was compared with the RI-CLPM with FIML on the present sample (*n* = 1560). Besides, RI-CLPMs across genders were constructed to test robustness. Developmentally sensitive analysis was conducted in two ways. First, respondents’ baseline age was treated as a continuous moderator in the RI-CLPM for the entire sample and the youth sample. Second, the RI-CLPM was tested in the subsample of emerging adults aged 18 to 24 years. The estimation of the RI-CLPM with a continuous moderator followed the parameter settings and procedures outlined in two methodological papers (Ozkok et al., 2022; Speyer et al., 2023) using a Bayesian estimator. A Potential Scale Reduction (PSR) value below 1.05 was used as the convergence criterion, with a PSR closer to 1 indicating good convergence of Markov Chain Monte Carlo simulations. To simplify computation, the moderating effect of age was only tested on significant cross-lagged and autoregressive paths in the RI-CLPM. The moderating effect for each path was constrained to be equal across waves. No covariates were included for the RI-CLPM with the moderator.

## Results

### Descriptive Statistics and Attrition Analysis

Table 1 presents the descriptive statistics for the three main variables and their pairwise correlations over time after FIML is applied. The present sample consisted of 1,560 young gamers (45.3% female, Mage = 23.38, SD = 3.81 years), comprising 875 youth (43.20% female, M_age_ = 20.52, SD = 2.38 years) and 685 adults (47.88% female, Mage = 27.03, SD = 1.42 years). The trait-like between-person effects explained 53%-62% of the variance in internet gaming disorder symptoms (ICC = 0.61), depressive symptoms (ICC = 0.62), and hikikomori symptoms (ICC = 0.53). Table S1 presents the attrition analysis of baseline measurements between the longitudinal sample with complete records and the dropout sample. It shows that dropouts tended to be younger male gamers with a higher risk of internet gaming disorder and hikikomori symptoms.

**Table 1:** Mean, standard deviation, and pair-wise correlations between the three main variables in the analytical sample (*n* = 1560)

	Mean	SD	1	2	3	4	5	6	7	8	9
1. T1 Internet gaming disorder symptoms	2.445	0.021	1.000								
2. T2 Internet gaming disorder symptoms	2.223	0.031	0.647^***^	1.000							
3. T3 Internet gaming disorder symptoms	2.221	0.036	0.598^***^	0.588^***^	1.000						
4. T1 Depressive symptoms	0.953	0.015	0.467^***^	0.354^***^	0.351^***^	1.000					
5. T2 Depressive symptoms	0.885	0.025	0.365^***^	0.429^***^	0.356^***^	0.646^***^	1.000				
6. T3 Depressive symptoms	0.875	0.026	0.357^***^	0.311^***^	0.404^***^	0.625^***^	0.604^***^	1.000			
7. T1 Hikikomori symptoms	1.588	0.039	0.464^***^	0.395^***^	0.360^***^	0.513^***^	0.403^***^	0.386^***^	1.000		
8. T2 Hikikomori symptoms	1.399	0.060	0.402^***^	0.371^***^	0.368^***^	0.393^***^	0.472^***^	0.413^***^	0.568^***^	1.000	
9. T3 Hikikomori symptoms	1.257	0.068	0.321^***^	0.306^***^	0.368^***^	0.393^***^	0.440^***^	0.467^***^	0.478^***^	0.526^***^	1.000

All missing values in the analytical sample were imputed using full information maximum likelihood (FIML)

T1 = Time 1, T2 = Time 2, T3 = Time 3

***p < 0.001

### RI-CLPM Results

Table 2 presents the model fit results with varying constraints. For the RI-CLPMs in both youth and adult gamers, Model 4 has relatively high CFI and TLI, and the smallest AIC and BIC. The RMSEA was relatively small, with the narrowest CIs, suggesting better estimation accuracy. Therefore, the RI-CLPM with autoregressive and cross-lagged paths, as well as covariance and variance constrained to be the same across time, was determined to be the more parsimonious model for two age groups. After adding covariates, the final model performed well (youth gamers: $\:{\chi\:}^{2}$ = 104.761, df = 84, CFI = 0.988, TLI = 0.980, RMSEA = 0.017; adult gamers: $\:{\chi\:}^{2}$ = 106.500, df = 84, CFI = 0.985, TLI = 0.965, RMSEA = 0.020).

**Table 2:** Model fits and comparison of RI-CLPMs with varying constraints

	χ^2^	df	ΔCFI	ΔTLI	RMSEA	CFI	TLI	AIC	BIC
Youth gamers									
Model 1	4.750	3	-	-	0.026	0.998	0.976	10089.157	10332.642
Model 2	15.469	6	0.009	0.040	0.042	0.989	0.936	10093.576	10332.739
Model 3	24.547	12	0.003	-0.022	0.035	0.986	0.958	10092.224	10292.742
Model 4	29.392	18	-0.001	-0.016	0.027	0.987	0.974	10088.297	10260.169
Model 5 (final)	104.761	84			0.017	0.988	0.980	9460.516	9789.937
Adult gamers									
Model 1	4.122	3	-	-	0.023	0.999	0.986	8187.775	8418.776
Model 2	5.562	6	0.000	-0.009	0.017	0.999	0.995	8184.797	8402.209
Model 3	19.607	12	0.007	0.019	0.030	0.992	0.976	8188.424	8378.660
Model 4	20.828	18	-0.005	-0.018	0.015	0.997	0.994	8178.638	8341.697
Model 5 (final)	106.500	84			0.020	0.985	0.976	7912.169	8224.699

Note. Model 1 is the baseline model with no constraints. Model 2 constrains the autoregressive effects to be equal across time. Model 3 constrains the autoregressive and cross-lagged effects to be equal across time. Model 4 constrains the autoregressive and cross-lagged effects, and the covariance of errors to be equal across time. Model 5 is the model 4 with covariates. Δ = value of the previous model – the value of the actual model

**Fig. 1:**
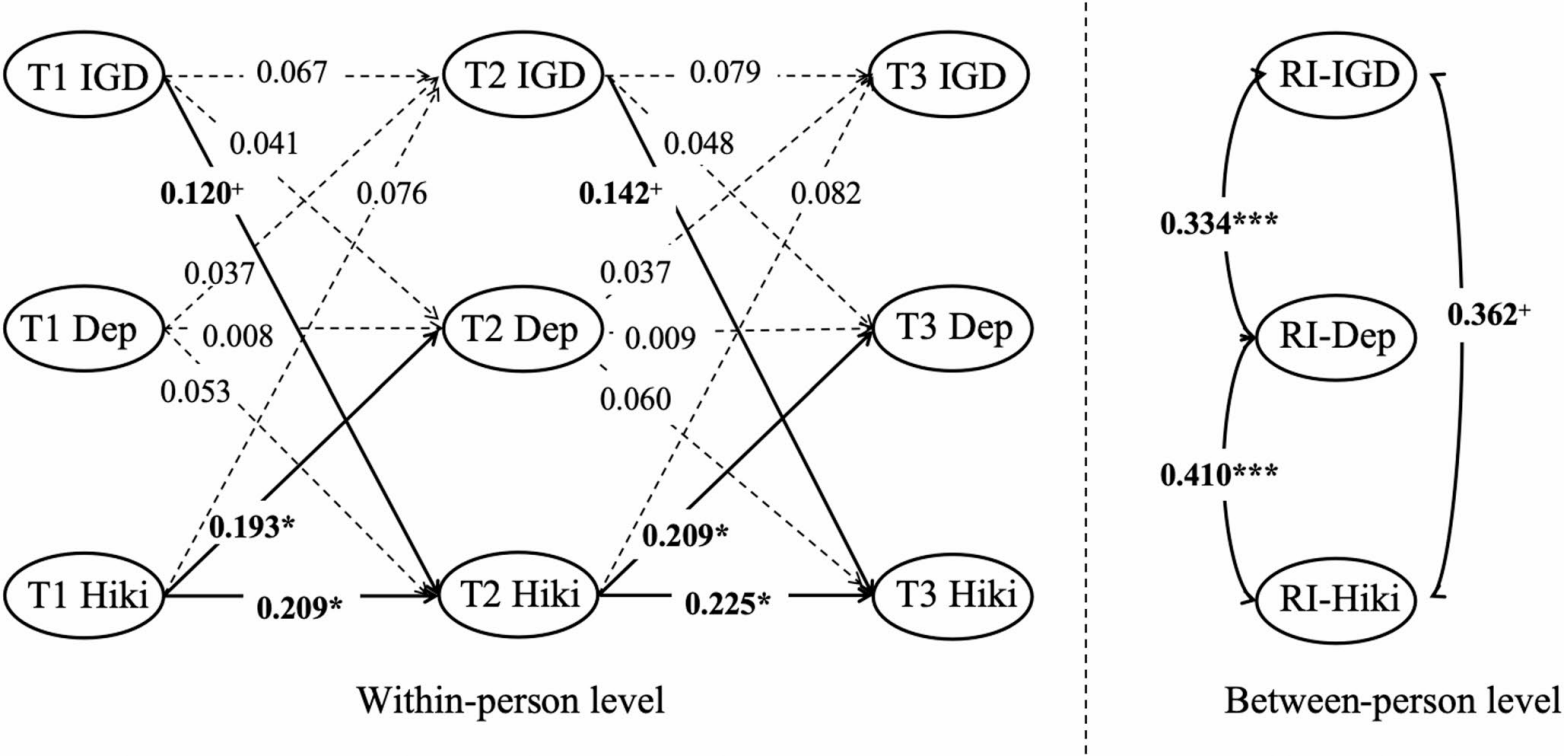
The RI-CLPM of internet gaming disorder symptoms, depressive symptoms, and hikikomori symptoms with covariates in youth gamers. Note. IGD = Internet Gaming Disorder symptoms; Dep = Depressive symptoms; Hiki = Hikikomori symptoms; RI = random intercept. T1 = Time 1, T2 = Time 2, T3 = Time 3. All estimated effects were standardized. Dashed lines represent nonsignificant effects. ^*+*^*p* < 0.1, *p < 0.05, **p < 0.01, ***p < 0.001

**Fig. 2:**
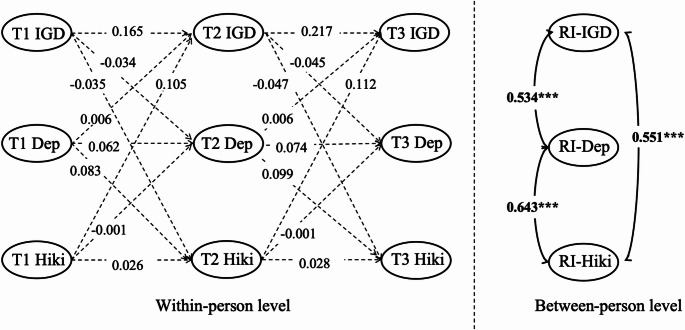
The RI-CLPM of internet gaming disorder symptoms, depressive symptoms, and hikikomori symptoms with covariates in adult gamers. Note. IGD = Internet Gaming Disorder symptoms; Dep = Depressive symptoms; Hiki = Hikikomori symptoms; RI = random intercept. T1 = Time 1, T2 = Time 2, T3 = Time 3. All estimated effects were standardized. Dashed lines represent nonsignificant effects. *p < 0.05, **p < 0.01, ***p < 0.001

At the within-person level, Fig. 1 and Table S3 show the positive cross-lagged effect of internet gaming disorder on hikikomori symptoms with marginal significance (T1 to T2: $\:\beta\:$ = 0.120, *p* = 0.070; T2 to T3: $\:\beta\:$ = 0.142, *p* = 0.070) and a significant path from hikikomori to depressive symptoms (T1 to T2: $\:\beta\:$ = 0.193, *p* = 0.034; T2 to T3: $\:\beta\:$ = 0.209, *p* = 0.034) among youth. No significant within-person paths were found between internet gaming disorder and hikikomori symptoms. For the autoregressive effects, only hikikomori symptoms predicted itself at the next time point significantly (T1 to T2: $\:\beta\:$ = 0.209, *p* = 0.037; T2 to T3: $\:\beta\:$ = 0.225, *p* = 0.037). For the RI-CLPM in adult gamers (shown in Fig. 2 and Table S4), no significant within-person effects have been observed between symptoms of internet gaming disorder, depression, and hikikomori.

At the between-person level, there were significant positive correlations between pairs of random intercepts of internet gaming disorder, depressive, and hikikomori symptoms among both youth (*r* = 0.334–0.410; *p* = 0.020–0.054) and adult gamers (*r* = 0.534–0.643; *p* < 0.001). Specifically, Table 3 displays the explanatory effects of covariates. Playing for escapism was positively associated with symptoms of internet gaming disorder, depression, and hikikomori among youth (B = 0.429, 0.243, 0.292; *p* < 0.001) and adults (B = 0.360, 0.252, 0.230; *p* < 0.001). Higher disruptor traits were linked to three constructs in adult gamers (B = 0.125–0.214; *p* < 0.01), whereas they were related to internet gaming disorder and hikikomori symptoms in youth (B = 0.117–0.251; *p* < 0.05). Engagement in multiplayer online battle arena games (B = 0.081–0.208; *p* < 0.01) and massively multiplayer online role-playing games (B = 0.101–0.187; *p* < 0.05) was associated with three constructs for youth, while playing multiplayer online battle arena games was only associated with internet gaming disorder (B = 0.150; *p* < 0.001), and playing massively multiplayer online role-playing games was associated with internet gaming disorder and hikikomori symptoms among adult gamers (B = 0.124–0.162; *p* < 0.01). Both youth (B = -0.320 - -0.176; *p* < 0.001) and adult gamers (B = -0.247 - -0.214; *p* < 0.001) less satisfied with their families reported more depressive and hikikomori symptoms.

**Table 3:** Associations between covariates and random intercepts of three variables in the final model

	Internet gaming disorder symptoms		Depressive symptoms		Hikikomori symptoms	
	standard B	*p*-value	standard B	*p*-value	standard B	*p*-value
Youth gamers (15–24)						
Females	-0.050	**<** 0.112	**0.129**	< 0.001**	0.011	**<** 0.783
Age	-0.028	**<** 0.368	-0.035	**<** 0.370	**-0.108**	< 0.009**
Monthly income	0.005	**<** 0.869	-0.052	**<** 0.169	-0.071	**<** 0.091
Family satisfaction	-0.044	**<** 0.198	**-0.320**	< 0.001***	**-0.176**	< 0.001***
Strategy games	0.038	**<** 0.229	0.031	**<** 0.442	0.109	**<** 0.013
First-person shooter games	**0.135**	< 0.001***	0.010	**<** 0.795	0.082	**<** 0.067
Multiplayer online battle arena games	**0.081**	< 0.001***	**0.119**	< 0.003**	**0.208**	< 0.001***
Massively multiplayer online role-playing games	**0.183**	< 0.013*	**0.101**	< 0.010*	**0.187**	< 0.001***
Sports games	**0.103**	< 0.003**	0.041	**<** 0.268	0.077	**<** 0.076
Disruptor	**0.251**	< 0.001***	0.085	**<** 0.056	**0.117**	< 0.014*
Escapism	**0.429**	< 0.001***	**0.243**	< 0.001***	**0.292**	< 0.001***
Adult gamers (25–29)						
Females	**-0.080**	< 0.043*	**0.102**	**< 0.014**	0.076	**<** 0.086
Age	0.049	**<** 0.185	-0.005	**<** 0.898	-0.026	**<** 0.546
Monthly income	-0.020	**<** 0.583	-0.054	**<** 0.173	**-0.093**	< 0.037*
Family satisfaction	-0.077	**<** 0.075	**-0.247**	**< 0.001**	**-0.214**	< 0.001***
Strategy games	0.068	**<** 0.077	**0.091**	**< 0.028**	**0.114**	< 0.010*
First-person shooter games	**0.097**	< 0.012*	-0.014	**<** 0.730	0.043	**<** 0.328
Multiplayer online battle arena games	**0.150**	< 0.001***	0.025	**<** 0.552	0.070	**<** 0.117
Massively multiplayer online role-playing games	**0.124**	< 0.001**	0.046	**<** 0.264	**0.162**	< 0.001***
Sports games	0.048	**<** 0.218	0.078	**<** 0.075	0.024	**<** 0.597
Disruptor	**0.214**	< 0.001***	**0.147**	**< 0.002**	**0.125**	< 0.006**
Escapism	**0.360**	< 0.001***	**0.252**	**< 0.001**	**0.230**	< 0.001***

Note. The bold refers to the significant results. *p < 0.05, **p < 0.01, ***p < 0.001

### Sensitivity Analysis

The RI-CLPM with FIML displays consistent results in the analytical sample (*n* = 1560; Table S5) as the complete-case RI-CLPM based on the sample without partial respondents (*n* = 408; Table S6). Both RI-CLPMs show no significant within-person effects but stable between-person associations. Autoregressive effects of hikikomori symptoms were larger in the RI-CLPM with FIML than in the RI-CLPM without FIML due to the larger sample size. For RI-CLPMs across gender (Tables S7 & S8), no differences in between-person associations or cross-lagged effects were observed. The autoregressive effects of internet gaming disorder symptoms were significant among girls.

Table S9 shows the results of the RI-CLPM with age as a continuous moderator on the significant cross-lagged paths identified earlier, including paths from internet gaming disorder symptoms to hikikomori symptoms, from hikikomori symptoms to depressive symptoms, and the autoregressive path of hikikomori symptoms. The RI-CLPM converged with 42,800 iterations and a PSR of 1.045. Age only had significant between-person associations, with no significant moderating effects on the cross-lagged and autoregressive paths. The model indicates no moderating effects across the entire sample aged 15 to 29 years. Similarly, for the youth sample, Table S10 shows the RI-CLPM with age as a continuous moderator in youth gamers aged 15 to 24 years. No significant moderating effects were found on within-person effects in the youth sample, suggesting the robustness of the results. Lastly, Table S11 shows that RI-CLPM results in emerging adults aged 18 to 24 years replicated those in the RI-CLPM among youth gamers. These findings demonstrate that the RI-CLPM results for both youth and adult gamers are robust.

## Discussion

Despite the substantial interrelations among internet gaming disorder, depressive, and hikikomori symptoms, the developmental directionality of these conditions remains unclear, given a lack of simultaneous longitudinal research. Previous mixed findings regarding the relationship between internet gaming disorder and depressive symptoms highlight the necessity to account for age differences and relevant covariates, and scarce longitudinal research involving hikikomori limits our understanding of how hikikomori symptoms are associated with internet gaming disorder and depressive symptoms over time. To address these gaps, this study employed the random intercept cross-lagged panel model (RI-CLPM) to examine the longitudinal within-person relations between these three conditions among young gamers in Hong Kong. By accounting for stable between-person differences and incorporating contextual factors related to family and internet gaming, this study clarified the developmental sequence of these constructs and provided a holistic view of the factors driving their co-occurrence.

In parallel with a previous RI-CLPM study (Marciano et al., 2022), most covariates included in this study had significant effects on random intercepts, providing a comprehensive understanding of the relations among internet gaming disorder, depressive, and hikikomori symptoms. There were consistent between-person associations in youth and adult gamers, but age differences in longitudinal within-person effects. The results indicate that: (i) no longitudinal within-person effects between internet gaming disorder and depressive symptoms in youth and adult gamers; (ii) internet gaming disorder symptoms positively predicted hikikomori symptoms in youth; (iii) hikikomori symptoms positively predicted depressive symptoms in youth; (iv) no longitudinal within-person effects in adults; (v) pairs of internet gaming disorder, depressive, and hikikomori symptoms had significant and positive between-person associations in both youth and adults; (vi) family satisfaction, playing different game genres, disruptor traits, and escapism motive were associated with random intercepts of three constructs. Generally, youth gaming behaviors and socio-emotional skills tend to develop and interact with one another as youth maturing executive functions and emotional systems, as well as their sensitivity to the environment and their need for interpersonal connections. For adult gamers with relatively stable identity development, most have begun working and are focusing more on employment and financial issues (Hatano et al., 2024). Refined cognitive skills enable them to rationally consider the influences of excessive gaming and prolonged isolation and to better control their behaviors and negative emotions.

Specifically, hikikomori symptoms significantly and positively predicted depressive symptoms in youth, but not vice versa or in adult gamers, consistent with previous research among college students (Liu et al., 2020). As socialization becomes increasingly important in youth development, hikikomori symptoms among youth gamers may reflect disturbances in social interaction, such as exclusion from peer groups and impaired interpersonal relationships. Therefore, loneliness and isolation related to social difficulties may contribute to depressive symptoms (Pupi et al., 2025). The insignificant path from depressive symptoms to hikikomori symptoms could be explained by the fact that hikikomori symptoms are associated with interpersonal problems in youth gamers and stressful life events in adults (Barzeva et al., 2019). This supports the claim that hikikomori symptoms may not be the secondary condition of depressive symptoms (Amendola, 2024). On the contrary, a recent study highlights depressive symptoms as a key risk factor for social withdrawal in early adolescents (Yang & Zhang, 2026). The discrepancy may be due to the older sample and subclinical measurements of hikikomori in the present study. At the between-person level, family dissatisfaction was robustly linked to depressive and hikikomori symptoms among both youth and adult gamers. Positive family relations are associated with better mental health from adolescence to midlife, as they promote the development of coping skills for managing negative emotions and cumulative stressors (Chen & Harris, 2019). Thus, intervention in early family life to foster positive youth development could be important to reduce the comorbidity of depressive and hikikomori symptoms.

Consistent with a weak predictive effect of internet gaming disorder symptoms on later psychosocial well-being (Teng et al., 2020), this study found a marginally significant cross-lagged path from internet gaming disorder to hikikomori symptoms among youth. This suggests that the social displacement theory may be more applicable to interpersonal problems among youth gamers, who may have underdeveloped cognitive control and be more sensitive to social rewards (Wang et al., 2024). Youth’s problematic gaming behaviors consume considerable time and energy, leaving less for their real-world lives. This coincides with previous findings that adolescents with internet gaming disorder symptoms were more likely to refuse school attendance and experience more interpersonal problems (Cheng et al., 2018). Preventing problematic gaming behaviors and developing healthy habits may help reduce hikikomori symptoms in youth.

The present findings revealed no longitudinal within-person effects between internet gaming disorder and depressive symptoms in either direction among youth or adult gamers, in contrast to reciprocal associations observed in previous CLPM studies (Dang et al., 2024; Teng et al., 2021; Wang et al., 2022). A plausible explanation is that these studies conflated the stable between-person effects, which have been identified as indispensable in RI-CLPM studies. The present results were consistent with the RI-CLPM studies in children (Hygen et al., 2020) and adolescents (Zhang et al., 2025) and extended to youth and adult gamers. Another explanation of non-significant within-person effects is that the bidirectional relations between internet gaming disorder and depressive symptoms may be influenced by common factors, such as gaming motives. The vicious cycle is more likely to occur when gamers relieve negative emotions through online games. This supports the common cause hypothesis that the co-occurrence of internet gaming disorder and depressive symptoms may be attributed to time-invariant differences.

The between-person associations among the random intercepts of internet gaming disorder, depressive, and hikikomori symptoms were significant for youth and adult gamers. For the covariates, the only common factor was the escapism motive, aligning with ‘gaming as escape’ as the bridge symptom in the cross-sectional network on symptoms of internet gaming disorder, depression, and hikikomori (Fong et al. 2024a, b). Considering the distinct factors across age groups, playing multiplayer online battle arena games and massively multiplayer online role-playing games was associated with higher risks of internet gaming disorder, depressive, and hikikomori symptoms among youth. Youth gamers may lack alternative means of coping but turn to a more accessible way to satisfy their unmet needs (Schneider et al., 2018). The multiplayer and online interaction elements of multiplayer online battle arena games and massively multiplayer online role-playing games can fulfill their needs for social compensation due to prolonged social isolation (Giardina et al., 2021). Additionally, the virtual environment and immersive setting may fascinate depressed gamers to play longer (Saini & Hodgins, 2023). For adult gamers, comorbidity was associated with elevated disruptor traits, similar to an impulsive/aggressive type of internet gaming disorder (Ko et al., 2023). The rule-breaking behaviors in online games may provide adults with a sense of achievement, especially when they are upset by real-world challenges and confused about adult responsibilities. A focus on predisposing personality traits and life issues may benefit adults with comorbidities of internet gaming disorder, depressive, and hikikomori symptoms.

### Limitations

While the present study extends previous research in several ways, a few limitations should be noted in this study. First, the current study did not account for all potential variables that might explain the co-occurrence of these conditions. Factors that may account for the co-occurrence, such as genetic etiology, were not considered. Future studies should conduct a comprehensive investigation of the common causes of comorbidity among internet gaming disorder, depressive, and hikikomori symptoms using a broader set of predisposing and contextual factors in the physiopsychological and behavioral domains.

Second, the demographic composition of the sample may limit the generalizability of the findings to other age groups. This study included only youth and adult gamers who play online games for at least 3 h per week. The within-person relations observed in this study appear to be specific to youth gamers, leaving the exact developmental differences across the lifespan only partially understood. Although the sensitivity analyses with age as the continuous moderator showed no moderating effect on the within-person effects, developmental differences across age may still exist. Future studies can model the dynamic relations between symptoms of internet gaming disorder, depression, and hikikomori in other age groups, such as children and early adolescents, or build an age-specific model to capture developmental changes.

Finally, a relatively high attrition rate and subsequent non-response bias may impact the overall robustness of the study. Specifically, respondents who were younger, male, and had higher baseline internet gaming disorder and hikikomori symptom levels exhibited higher attrition rates. Although financial incentives were adjusted to provide an e-voucher upon survey completion (which likely helped lower attrition at T3), future studies should maintain closer connections with participants to ensure low attrition throughout the longitudinal process.

## Conclusion

Although the comorbidity of internet gaming disorder, depressive, and hikikomori symptoms is increasingly recognized, the precise developmental mechanisms driving this co-occurrence remain largely unexplored. This is particularly the case whether these conditions interact as dynamic, transient states or stable, entrenched traits across different age groups. By separating longitudinal within-person and between-person effects, the present study addressed this gap and revealed developmental differences between youth and adult gamers. Specifically, problematic gaming behaviors and hikikomori symptoms act as predictive antecedents for hikikomori and depressive symptoms, accordingly, driving longitudinal within-person interactions that exacerbate comorbidity in youth. Conversely, among young adults, the co-occurrence of these conditions is largely due to stable, trait-like between-person differences rather than to prospective within-person changes. Furthermore, the stable between-person associations are heavily influenced by contextual factors within the family (e.g., family dissatisfaction) and gaming domains (e.g., game genres and motives). These findings advance the understanding of early adulthood by proving that the fundamental drivers of this comorbidity shift from acute, state-like vulnerabilities in youth to stable, trait-level differences in adulthood. Ultimately, this research provides a critical developmental framework, underscoring how age and context fundamentally dictate the longitudinal relations between internet gaming disorder, depressive, and hikikomori symptoms.

## Supplementary Information

Below is the link to the electronic supplementary material.

Supplementary Material 1

## Data Availability

The datasets generated and/or analyzed during the current study are not publicly available but are available from the corresponding author on reasonable request.
