# Supplementary material for: Longitudinal Relations Between Internet Gaming Disorder Symptoms, Depressive Symptoms, and Hikikomori Symptoms Among Young Gamers – Random Intercept Cross-Lagged Panel Model With Contextual Factors
Source: J Youth Adolesc. 2026 Apr 15;55(7):1764–76. doi: 10.1007/s10964-026-02352-7 (PMC13328286; doi:10.1007/s10964-026-02352-7)
Supplement: Supplementary file 1 — Supplementary Material 1 [file 10964_2026_2352_MOESM1_ESM.docx]

**Supplementary materials**

**Longitudinal Relations Between Internet Gaming Disorder Symptoms, Depressive Symptoms, and Hikikomori Symptoms Among Young Gamers – Random Intercept Cross-Lagged Panel Model With Contextual Factors**

Table S1. Attrition analysis on the longitudinal sample and the dropout sample.

Table S2. Longitudinal measurement invariance tests for main variables.

Table S3. Parameter estimates for the RI-CLPM using full-information maximum likelihood with covariates in youth gamers.

Table S4. Parameter estimates for the RI-CLPM using full-information maximum likelihood with covariates in adult gamers.

Table S5. Parameter estimates for the RI-CLPM using full-information maximum likelihood with covariates in the whole analytical sample.

Table S6. Parameter estimates for the RI-CLPM with covariates in the longitudinal sample with complete records using listwise deletion.

Table S7. Parameter estimates for the RI-CLPM using full-information maximum likelihood with covariates among boys.

Table S8. Parameter estimates for the RI-CLPM using full-information maximum likelihood with covariates among girls.

Table S9. Parameter estimates for the RI-CLPM using full-information maximum likelihood with age as the continuous moderator in the whole analytical sample.

Table S10. Parameter estimates for the RI-CLPM using full-information maximum likelihood with age as the continuous moderator in youth gamers.

Table S11. Parameter estimates for the RI-CLPM using full-information maximum likelihood with covariates in emerging adult gamers aged 18 to 24.

Table S1. Attrition analysis on the longitudinal sample and the dropout sample

| N = 1560 | Longitudinal sample with complete records  Mean (SD) / n (%) | Dropouts  Mean (SD) / n (%) | statistics | p-value |
| --- | --- | --- | --- | --- |
| N | 408 | 1152 |  |  |
| N_Youth (15 - 24)_ | 209 (51.00%) | 666 (58.00%) | 5.04 | < 0.05 |
| Gender (male) | 192 (47.06%) | 662 (57.47%) | 12.75 | < 0.01 |
| Age | 23.95 (3.49) | 23.18 (3.90) | 3.75 | < 0.01 |
| Internet gaming disorder symptoms | 2.27 (0.78) | 2.51 (0.84) | -5.18 | < 0.01 |
| Depressive symptoms | 0.91 (0.61) | 0.94 (0.57) | -0.89 | 0.37 |
| Hikikomori symptoms | 1.40 (1.52) | 1.65 (1.56) | -2.83 | < 0.01 |

Note. Baseline measurements were used.

Table S2. Longitudinal measurement invariance tests for main variables.

|  | *χ^2^ (df)* | ΔCFI | CFI | RMSEA | SRMR | BIC |
| --- | --- | --- | --- | --- | --- | --- |
| Internet gaming disorder symptoms |  |  |  |  |  |  |
| Configural | 666.569 (294) | - | 0.961 | 0.029 | 0.042 | 60797.849 |
| Weak | 695.582 (310) | 0.001 | 0.960 | 0.028 | 0.047 | 60706.990 |
| Strong | 749.581 (328) | 0.004 | 0.956 | 0.029 | 0.055 | 60623.710 |
|  |  |  |  |  |  |  |
| Depressive symptoms |  |  |  |  |  |  |
| Configural | 651.780 (294) | - | 0.953 | 0.028 | 0.042 | 52028.011 |
| Weak | 668.946 (310) | 0.000 | 0.953 | 0.027 | 0.044 | 51931.920 |
| Strong | 725.152 (328) | 0.006 | 0.947 | 0.028 | 0.046 | 51861.071 |
|  |  |  |  |  |  |  |
| Hikikomori symptoms |  |  |  |  |  |  |
| Configural | 215.158 (72) | - | 0.930 | 0.036 | 0.047 | 13686.769 |
| Weak | 229.924 (80) | 0.003 | 0.927 | 0.035 | 0.051 | 13644.689 |
| Strong | 264.278 (90) | 0.008 | 0.919 | 0.035 | 0.057 | 13608.988 |

Note. Δ= value of the previous model – the value of the actual model.

Table S3. Parameter estimates for the RI-CLPM using full-information maximum likelihood with covariates in youth gamers (n = 875).

| Parameter | Standard beta | Standard error | p-value |
| --- | --- | --- | --- |
| Between-person effects |  |  |  |
| **IGD ↔ Dep** | **0.334** | **0.021** | **0.020** |
| **IGD ↔ Hiki** | **0.362** | **0.057** | **0.054** |
| **Dep ↔ Hiki** | **0.410** | **0.052** | **0.028** |
| Cross-lagged effects |  |  |  |
| T1 IGD → T2 Dep | 0.041 | 0.070 | 0.628 |
| **T1 IGD → T2 Hiki** | **0.120** | **0.165** | **0.070** |
| T2 IGD → T3 Dep | 0.048 | 0.070 | 0.628 |
| **T2 IGD → T3 Hiki** | **0.142** | **0.165** | **0.070** |
| T1 Dep → T2 IGD | 0.032 | 0.178 | 0.767 |
| T1 Dep → T2 Hiki | 0.053 | 0.308 | 0.556 |
| T2 Dep → T3 IGD | 0.037 | 0.178 | 0.767 |
| T2 Dep → T3 Hiki | 0.060 | 0.308 | 0.556 |
| T1 Hiki → T2 IGD | 0.076 | 0.045 | 0.382 |
| **T1 Hiki → T2 Dep** | **0.193** | **0.033** | **0.034** |
| T2 Hiki → T3 IGD | 0.082 | 0.045 | 0.382 |
| **T2 Hiki → T3 Dep** | **0.209** | **0.033** | **0.034** |
| Autoregressive effects |  |  |  |
| T1 IGD → T2 IGD | 0.067 | 0.163 | 0.625 |
| T2 IGD → T3 IGD | 0.079 | 0.163 | 0.625 |
| T1 Dep → T2 Dep | 0.008 | 0.131 | 0.942 |
| T2 Dep → T3 Dep | 0.009 | 0.131 | 0.942 |
| **T1 Hiki → T2 Hiki** | **0.209** | **0.109** | **0.037** |
| **T2 Hiki → T3 Hiki** | **0.225** | **0.109** | **0.037** |

Note. IGD = Internet Gaming Disorder symptoms; Dep = Depressive symptoms; Hiki = Hikikomori symptoms. Covariates included age, gender, game genres, family income, family satisfaction, disruptor traits, and escapism motive.

Table S4. Parameter estimates for the RI-CLPM using full-information maximum likelihood with covariates in adult gamers (n = 685).

| Parameter | Standard beta | Standard error | p-value |
| --- | --- | --- | --- |
| Between-person effects |  |  |  |
| **IGD ↔ Dep** | **0.534** | **0.018** | **<0.001** |
| **IGD ↔ Hiki** | **0.551** | **0.047** | **<0.001** |
| **Dep ↔ Hiki** | **0.643** | **0.036** | **<0.001** |
| Cross-lagged effects |  |  |  |
| T1 IGD → T2 Dep | -0.034 | 0.086 | 0.730 |
| T1 IGD → T2 Hiki | -0.035 | 0.217 | 0.689 |
| T2 IGD → T3 Dep | -0.045 | 0.086 | 0.730 |
| T2 IGD → T3 Hiki | -0.047 | 0.217 | 0.689 |
| T1 Dep → T2 IGD | 0.006 | 0.161 | 0.950 |
| T1 Dep → T2 Hiki | 0.083 | 0.276 | 0.311 |
| T2 Dep → T3 IGD | 0.006 | 0.161 | 0.950 |
| T2 Dep → T3 Hiki | 0.099 | 0.276 | 0.311 |
| T1 Hiki → T2 IGD | 0.105 | 0.055 | 0.265 |
| T1 Hiki → T2 Dep | -0.001 | 0.031 | 0.987 |
| T2 Hiki → T3 IGD | 0.112 | 0.055 | 0.265 |
| T2 Hiki → T3 Dep | -0.001 | 0.031 | 0.987 |
| Autoregressive effects |  |  |  |
| T1 IGD → T2 IGD | 0.165 | 0.191 | 0.248 |
| T2 IGD → T3 IGD | 0.217 | 0.191 | 0.248 |
| T1 Dep → T2 Dep | 0.062 | 0.143 | 0.602 |
| T2 Dep → T3 Dep | 0.074 | 0.143 | 0.602 |
| T1 Hiki → T2 Hiki | 0.026 | 0.116 | 0.806 |
| T2 Hiki → T3 Hiki | 0.028 | 0.116 | 0.806 |

Note. IGD = Internet Gaming Disorder symptoms; Dep = Depressive symptoms; Hiki = Hikikomori symptoms. Covariates included age, gender, game genres, family income, family satisfaction, disruptor traits, and escapism motive.

Table S5. Parameter estimates for the RI-CLPM using full-information maximum likelihood with covariates in the whole sample (n = 1560).

| Parameter | Standard beta | Standard error | p-value |
| --- | --- | --- | --- |
| Between-person effects |  |  |  |
| **IGD ↔ Dep** | **0.460** | **0.013** | **< 0.001** |
| **IGD ↔ Hiki** | **0.474** | **0.034** | **< 0.001** |
| **Dep ↔ Hiki** | **0.563** | **0.029** | **< 0.001** |
| Cross-lagged effects |  |  |  |
| T1 IGD → T2 Dep | 0.002 | 0.053 | 0.976 |
| T1 IGD → T2 Hiki | 0.066 | 0.129 | 0.202 |
| T2 IGD → T3 Dep | 0.002 | 0.053 | 0.976 |
| T2 IGD → T3 Hiki | 0.082 | 0.129 | 0.202 |
| T1 Dep → T2 IGD | 0.008 | 0.117 | 0.905 |
| T1 Dep → T2 Hiki | 0.044 | 0.211 | 0.472 |
| T2 Dep → T3 IGD | 0.009 | 0.117 | 0.905 |
| T2 Dep → T3 Hiki | 0.051 | 0.211 | 0.472 |
| T1 Hiki → T2 IGD | 0.085 | 0.032 | 0.149 |
| T1 Hiki → T2 Dep | 0.093 | 0.023 | 0.131 |
| T2 Hiki → T3 IGD | 0.093 | 0.032 | 0.149 |
| T2 Hiki → T3 Dep | 0.102 | 0.023 | 0.131 |
| Autoregressive effects |  |  |  |
| T1 IGD → T2 IGD | 0.108 | 0.119 | 0.255 |
| T2 IGD → T3 IGD | 0.134 | 0.119 | 0.255 |
| T1 Dep → T2 Dep | 0.026 | 0.092 | 0.741 |
| T2 Dep → T3 Dep | 0.030 | 0.092 | 0.741 |
| **T1 Hiki → T2 Hiki** | **0.147** | **0.079** | **0.043** |
| **T2 Hiki → T3 Hiki** | **0.160** | **0.079** | **0.043** |

Note. IGD = Internet Gaming Disorder symptoms; Dep = Depressive symptoms; Hiki = Hikikomori symptoms. Covariates included age, gender, game genres, family income, family satisfaction, disruptor traits, and escapism motive.

Table S6. Parameter estimates for the RI-CLPM with covariates in the whole sample with complete records using listwise deletion (n = 408).

| Parameter | Standard beta | Standard error | p-value |
| --- | --- | --- | --- |
| Between-person effects |  |  |  |
| **IGD ↔ Dep** | **0.419** | **0.019** | **< 0.001** |
| **IGD ↔ Hiki** | **0.484** | **0.046** | **< 0.001** |
| **Dep ↔ Hiki** | **0.590** | **0.043** | **< 0.001** |
| Cross-lagged effects |  |  |  |
| T1 IGD → T2 Dep | 0.007 | 0.060 | 0.926 |
| T1 IGD → T2 Hiki | 0.038 | 0.135 | 0.490 |
| T2 IGD → T3 Dep | 0.007 | 0.060 | 0.926 |
| T2 IGD → T3 Hiki | 0.043 | 0.135 | 0.490 |
| T1 Dep → T2 IGD | 0.004 | 0.124 | 0.964 |
| T1 Dep → T2 Hiki | 0.061 | 0.212 | 0.368 |
| T2 Dep → T3 IGD | 0.004 | 0.124 | 0.964 |
| T2 Dep → T3 Hiki | 0.066 | 0.212 | 0.368 |
| T1 Hiki → T2 IGD | 0.084 | 0.034 | 0.222 |
| T1 Hiki → T2 Dep | 0.129 | 0.025 | 0.055 |
| T2 Hiki → T3 IGD | 0.090 | 0.034 | 0.222 |
| T2 Hiki → T3 Dep | 0.139 | 0.025 | 0.055 |
| Autoregressive effects |  |  |  |
| T1 IGD → T2 IGD | 0.059 | 0.128 | 0.600 |
| T2 IGD → T3 IGD | 0.067 | 0.128 | 0.600 |
| T1 Dep → T2 Dep | 0.003 | 0.099 | 0.977 |
| T2 Dep → T3 Dep | 0.003 | 0.099 | 0.977 |
| T1 Hiki → T2 Hiki | 0.144 | 0.081 | 0.056 |
| T2 Hiki → T3 Hiki | 0.155 | 0.081 | 0.056 |

Note. IGD = Internet Gaming Disorder symptoms; Dep = Depressive symptoms; Hiki = Hikikomori symptoms. Covariates included age, gender, game genres, family income, family satisfaction, disruptor traits, and escapism motive.

Table S7. Parameter estimates for the RI-CLPM using full-information maximum likelihood with covariates among boys (n = 854).

| Parameter | Standard beta | Standard error | p-value |
| --- | --- | --- | --- |
| Between-person effects |  |  |  |
| **IGD ↔ Dep** | **0.571** | **0.018** | **< 0.001** |
| **IGD ↔ Hiki** | **0.492** | **0.048** | **< 0.001** |
| **Dep ↔ Hiki** | **0.555** | **0.043** | **< 0.001** |
| Cross-lagged effects |  |  |  |
| T1 IGD → T2 Dep | -0.050 | 0.076 | 0.575 |
| T1 IGD → T2 Hiki | 0.133 | 0.175 | 0.064 |
| T2 IGD → T3 Dep | -0.065 | 0.076 | 0.575 |
| T2 IGD → T3 Hiki | 0.133 | 0.175 | 0.064 |
| T1 Dep → T2 IGD | -0.015 | 0.193 | 0.889 |
| T1 Dep → T2 Hiki | 0.046 | 0.311 | 0.628 |
| T2 Dep → T3 IGD | -0.018 | 0.193 | 0.889 |
| T2 Dep → T3 Hiki | 0.052 | 0.311 | 0.628 |
| T1 Hiki → T2 IGD | 0.123 | 0.050 | 0.154 |
| T1 Hiki → T2 Dep | 0.155 | 0.033 | 0.078 |
| T2 Hiki → T3 IGD | 0.133 | 0.050 | 0.154 |
| T2 Hiki → T3 Dep | 0.167 | 0.050 | 0.078 |
| Autoregressive effects |  |  |  |
| T1 IGD → T2 IGD | 0.038 | 0.170 | 0.770 |
| T2 IGD → T3 IGD | 0.049 | 0.170 | 0.770 |
| T1 Dep → T2 Dep | -0.006 | 0.159 | 0.963 |
| T2 Dep → T3 Dep | -0.007 | 0.159 | 0.963 |
| T1 Hiki → T2 Hiki | 0.111 | 0.115 | 0.297 |
| T2 Hiki → T3 Hiki | 0.119 | 0.115 | 0.297 |

Note. IGD = Internet Gaming Disorder symptoms; Dep = Depressive symptoms; Hiki = Hikikomori symptoms. Covariates included age, game genres, family income, family satisfaction, disruptor traits, and escapism motive.

Table S8. Parameter estimates for the RI-CLPM using full-information maximum likelihood with covariates among girls (n = 706).

| Parameter | Standard beta | Standard error | p-value |
| --- | --- | --- | --- |
| Between-person effects |  |  |  |
| **IGD ↔ Dep** | **0.326** | **0.019** | **0.006** |
| **IGD ↔ Hiki** | **0.477** | **0.051** | **0.001** |
| **Dep ↔ Hiki** | **0.596** | **0.041** | **< 0.001** |
| Cross-lagged effects |  |  |  |
| T1 IGD → T2 Dep | 0.091 | 0.064 | 0.243 |
| T1 IGD → T2 Hiki | -0.014 | 0.188 | 0.855 |
| T2 IGD → T3 Dep | 0.108 | 0.064 | 0.243 |
| T2 IGD → T3 Hiki | -0.016 | 0.188 | 0.855 |
| T1 Dep → T2 IGD | 0.065 | 0.114 | 0.343 |
| T1 Dep → T2 Hiki | 0.046 | 0.277 | 0.567 |
| T2 Dep → T3 IGD | 0.074 | 0.114 | 0.343 |
| T2 Dep → T3 Hiki | 0.052 | 0.277 | 0.567 |
| T1 Hiki → T2 IGD | 0.024 | 0.037 | 0.730 |
| T1 Hiki → T2 Dep | 0.024 | 0.029 | 0.756 |
| T2 Hiki → T3 IGD | 0.026 | 0.037 | 0.730 |
| T2 Hiki → T3 Dep | 0.027 | 0.029 | 0.756 |
| Autoregressive effects |  |  |  |
| **T1 IGD → T2 IGD** | **0.242** | **0.122** | **0.018** |
| **T2 IGD → T3 IGD** | **0.284** | **0.122** | **0.018** |
| T1 Dep → T2 Dep | 0.075 | 0.101 | 0.393 |
| T2 Dep → T3 Dep | 0.086 | 0.101 | 0.393 |
| T1 Hiki → T2 Hiki | 0.184 | 0.111 | 0.066 |
| T2 Hiki → T3 Hiki | 0.203 | 0.111 | 0.066 |

Note. IGD = Internet Gaming Disorder symptoms; Dep = Depressive symptoms; Hiki = Hikikomori symptoms. Covariates included age, game genres, family income, family satisfaction, disruptor traits, and escapism motive.

Table S9. Parameter estimates for the RI-CLPM using full-information maximum likelihood with age as the continuous moderator in the whole analytical sample (n = 1560).

| Parameter | Beta (95% CI) | Posterior S.D. |
| --- | --- | --- |
| Between-person effects |  |  |
| **IGD ↔ Dep** | **0.172 (0.108, 0.217)** | **0.028** |
| **IGD ↔ Hiki** | **0.392 (0.231, 0.511)** | **0.073** |
| **Dep ↔ Hiki** | **0.312 (0.181, 0.401)** | **0.057** |
| Cross-lagged effects |  |  |
| T1 IGD → T2 Dep | -0.216 (-0.560, 0.170) | 0.176 |
| T1 IGD → T2 Hiki | 0.377 (-3.708, 4.763) | 2.092 |
| T2 IGD → T3 Dep | -0.216 (-0.560, 0.170) | 0.176 |
| T2 IGD → T3 Hiki | 0.377 (-3.708, 4.763) | 2.092 |
| T1 Dep → T2 IGD | 0.079 (-2.685, 3.214) | 1.469 |
| T1 Dep → T2 Hiki | 1.629 (-2.679, 6.873) | 2.378 |
| T2 Dep → T3 IGD | 0.079 (-2.685, 3.214) | 1.469 |
| T2 Dep → T3 Hiki | 1.629 (-2.679, 6.873) | 2.378 |
| T1 Hiki → T2 IGD | 0.035 (-0.204, 0.253) | 0.115 |
| T1 Hiki → T2 Dep | -0.060 (-0.267, 0.153) | 0.110 |
| T2 Hiki → T3 IGD | 0.035 (-0.204, 0.253) | 0.115 |
| T2 Hiki → T3 Dep | -0.060 (-0.267, 0.153) | 0.110 |
| Autoregressive effects |  |  |
| T1 IGD → T2 IGD | 0.185 (-0.677, 0.922) | 0.416 |
| T2 IGD → T3 IGD | 0.185 (-0.677, 0.922) | 0.416 |
| T1 Dep → T2 Dep | 1.105 (-0.362, 1.946) | 0.566 |
| T2 Dep → T3 Dep | 1.105 (-0.362, 1.946) | 0.566 |
| T1 Hiki → T2 Hiki | 0.148 (-0.769, 1.082) | 0.465 |
| T2 Hiki → T3 Hiki | 0.148 (-0.769, 1.082) | 0.465 |
| Moderating effects of age |  |  |
| T1 IGD → T2 Hiki | -0.020 (-0.212, 0.054) | 0.090 |
| T2 IGD → T3 Hiki | -0.020 (-0.212, 0.054) | 0.090 |
| T1 Hiki → T2 Dep | 0.001 (-0.007, 0.009) | 0.004 |
| T2 Hiki → T3 Dep | 0.001 (-0.007, 0.009) | 0.004 |
| T1 Hiki → T2 Hiki | -0.004 (-0.038, 0.029) | 0.017 |
| T2 Hiki → T3 Hiki | -0.004 (-0.038, 0.029) | 0.017 |

Note. The RI-CLPM with a continuous moderator has been estimated using a Bayesian estimator. The significance of paths depends on whether the confidence interval covers 0 or not, instead of p-value given by MLR.

Table S10. Parameter estimates for the RI-CLPM using full-information maximum likelihood with age as the continuous moderator in youth gamers (n = 875).

| Parameter | Beta (95% CI) | Posterior S.D. |
| --- | --- | --- |
| Between-person effects |  |  |
| **IGD ↔ Dep** | **0.163 (0.100, 0.210)** | **0.029** |
| **IGD ↔ Hiki** | **0.387 (0.140, 0.539)** | **0.103** |
| **Dep ↔ Hiki** | **0.288 (0.082, 0.391)** | **0.082** |
| Cross-lagged effects |  |  |
| T1 IGD → T2 Dep | -0.209 (-0.634, 0.243) | 0.219 |
| T1 IGD → T2 Hiki | -5.305 (-13.955, 2.231) | 4.232 |
| T2 IGD → T3 Dep | -0.209 (-0.634, 0.243) | 0.219 |
| T2 IGD → T3 Hiki | -5.305 (-13.955, 2.231) | 4.232 |
| T1 Dep → T2 IGD | 0.557 (-1.604,4.148) | 1.480 |
| T1 Dep → T2 Hiki | 0.595 (-3.847, 5.023) | 2.280 |
| T2 Dep → T3 IGD | 0.557 (-1.604,4.148) | 1.480 |
| T2 Dep → T3 Hiki | 0.595 (-3.847, 5.023) | 2.280 |
| T1 Hiki → T2 IGD | 0.001 (-0.253, 0.210) | 0.120 |
| T1 Hiki → T2 Dep | 0.124 (-0.404, 0.756) | 0.255 |
| T2 Hiki → T3 IGD | 0.001 (-0.253, 0.210) | 0.120 |
| T2 Hiki → T3 Dep | 0.124 (-0.404, 0.756) | 0.255 |
| Autoregressive effects |  |  |
| T1 IGD → T2 IGD | -0.291 (-1.238, 0.506) | 0.446 |
| T2 IGD → T3 IGD | -0.291 (-1.238, 0.506) | 0.446 |
| T1 Dep → T2 Dep | 0.749 (-0.0887, 2.105) | 0.730 |
| T2 Dep → T3 Dep | 0.749 (-0.0887, 2.105) | 0.730 |
| T1 Hiki → T2 Hiki | 1.538 (-0.221, 3.390) | 0.905 |
| T2 Hiki → T3 Hiki | 1.538 (-0.221, 3.390) | 0.905 |
| Moderating effects of age |  |  |
| T1 IGD → T2 Hiki | 0.279 (-0.072, 0.696) | 0.203 |
| T2 IGD → T3 Hiki | 0.279 (-0.072, 0.696) | 0.203 |
| T1 Hiki → T2 Dep | -0.005 (-0.032, 0.019) | 0.011 |
| T2 Hiki → T3 Dep | -0.005 (-0.032, 0.019) | 0.011 |
| T1 Hiki → T2 Hiki | -0.066 (-0.149, 0.011) | 0.040 |
| T2 Hiki → T3 Hiki | -0.066 (-0.149, 0.011) | 0.040 |

Note. The RI-CLPM with a continuous moderator has been estimated using a Bayesian estimator. The significance of paths depends on whether the confidence interval covers 0 or not, instead of p-value given by MLR.

Table S11. Parameter estimates for the RI-CLPM using full-information maximum likelihood with covariates among emerging adults aged 18 to 24.

| Parameter | Standard beta | Standard error | p-value |
| --- | --- | --- | --- |
| Between-person effects |  |  |  |
| **IGD ↔ Dep** | **0.330** | **0.022** | **0.024** |
| **IGD ↔ Hiki** | **0.401** | **0.057** | **0.031** |
| **Dep ↔ Hiki** | **0.439** | **0.050** | **0.012** |
| Cross-lagged effects |  |  |  |
| T1 IGD → T2 Dep | 0.048 | 0.072 | 0.598 |
| T1 IGD → T2 Hiki | 0.114 | 0.165 | 0.092 |
| T2 IGD → T3 Dep | 0.056 | 0.072 | 0.598 |
| T2 IGD → T3 Hiki | 0.134 | 0.165 | 0.092 |
| T1 Dep → T2 IGD | 0.017 | 0.187 | 0.877 |
| T1 Dep → T2 Hiki | 0.027 | 0.322 | 0.744 |
| T2 Dep → T3 IGD | 0.020 | 0.187 | 0.877 |
| T2 Dep → T3 Hiki | 0.030 | 0.322 | 0.744 |
| T1 Hiki → T2 IGD | 0.085 | 0.045 | 0.326 |
| T1 Hiki → T2 Dep | 0.184 | 0.034 | 0.055 |
| T2 Hiki → T3 IGD | 0.092 | 0.045 | 0.326 |
| T2 Hiki → T3 Dep | 0.184 | 0.034 | 0.055 |
| Autoregressive effects |  |  |  |
| T1 IGD → T2 IGD | 0.071 | 0.167 | 0.616 |
| T2 IGD → T3 IGD | 0.084 | 0.167 | 0.616 |
| T1 Dep → T2 Dep | -0.003 | 0.145 | 0.979 |
| T2 Dep → T3 Dep | -0.004 | 0.145 | 0.979 |
| T1 Hiki → T2 Hiki | 0.200 | 0.111 | 0.053 |
| T2 Hiki → T3 Hiki | 0.213 | 0.111 | 0.053 |

Note. IGD = Internet Gaming Disorder symptoms; Dep = Depressive symptoms; Hiki = Hikikomori symptoms. Covariates included age, game genres, family income, family satisfaction, disruptor traits, and escapism motive.
